# The economic burden and costs of suicide and self-harm in Sweden

**DOI:** 10.1186/s12889-026-27156-z

**Published:** 2026-04-07

**Authors:** Inna Feldman, Camilla Nystrand, Johan Bjureberg, Gergö Hadlaczky

**Affiliations:** 1 Department of Public Health and Caring Sciences, UHE , Uppsala University, Uppsala, Sweden; 2 Department of Epidemiology and Global Health, Umeå University, Umeå, Sweden; 3 National center for suicide research and prevention (NASP) LIME, Karolinska Institute, Stockholm, Sweden; 4 Center for Health Economics, Informatics and Healthcare Research, Health Care Services Stockholm County (SLSO), Region Stockholm, Stockholm, Sweden; 5 Department of Clinical Neuroscience, Centre for Psychiatry Research, Karolinska Institutet, & Stockholm Health Care Services, Region Stockholm, Stockholm, Sweden

**Keywords:** Suicide, Self-harm, Economic burden

## Abstract

**Background:**

Suicide and self-harm represent a major public health challenge with significant human and economic consequences. Quantifying its economic burden is essential for guiding preventive strategies and resource allocation.

**Methods:**

This study estimated the societal costs of suicide and self-harm in Sweden in 2022 using data from high-quality national registries and publicly available sources. Costs encompassed direct costs (healthcare, emergency services, property damage) and indirect costs calculated using the human capital approach (productivity losses due to morbidity and premature death). Additionally, a monetary value for disability and death based on incidence data was used to monetize the burden of disease.

**Results:**

The total annual economic burden of suicide and self-harm in Sweden was substantial, amounting to approximately €970 million. Productivity losses from paid work represented the largest cost component, accounting for 67% of total costs, highlighting the considerable impact on the working-age population. When the monetary value of premature death and disability was estimated separately using a health-based valuation approach, the total burden increased to approximately €2.7 billion. Given the conservative assumptions applied in several components of the analysis, these estimates are likely to understate the true societal burden of suicide and self-harm.

**Conclusions:**

Suicide and self-harm impose a significant economic burden on Swedish society. Strengthening mental health services, workplace prevention programs, and postvention support could reduce both human suffering and societal costs. These findings provide valuable evidence to inform policymakers and stakeholders in designing cost-effective prevention strategies.

**Supplementary Information:**

The online version contains supplementary material available at 10.1186/s12889-026-27156-z.

## Introduction

Suicide stands as a serious public health issue. In the latest report from the WHO, it is estimated that 727,000 people died by suicide in 2021, representing a global suicide rate of 8.9 per 100,000 inhabitants [1]. In terms of burden of disease, the Global Burden of Disease Study (GBD) estimated suicide as the 22nd leading cause of Years of Life Lost (YLL) in 2019 [2]. In total, suicide is responsible for approximately 35 million years of life lost annually [3].

Over 1 200 people die by suicide each year in Sweden, representing an annual suicide rate of 12 per 100,000 individuals [4], surpassing both the global and European averages of 8.9 and 10.5, respectively. Additionally, suicide was the second leading cause of death in young people aged 15–19 years, after road injury and interpersonal violence, 5.53 per 100,000 individuals [4]. Furthermore, it is likely that suicide is under-reported [5] and this is especially true for suicidal acts with non-fatal outcomes, commonly known as suicide attempts or self-harm [1]. Suicidal behaviors produced approximately 87,000 Disability Adjusted Life Years (DALYs) in 2021 as the 7^Th^ top cause of DALYs in Sweden [6]. During the pandemic, suicide generated four times more YLL for the Swedish population than COVID-19 deaths did [7], given that COVID-19 mortality was greater in elderly population.

Every suicide is a tragedy, with far reaching impact on families, friends and communities. Previous estimates suggest that about six people are left behind following every suicide [8], but a recent study conducted in the US [9] estimated that for each suicide, 135 people are exposed to the death. In Sweden, one in two people knows someone who has died in suicide [9]. While the emotional costs of suicidal behaviors are difficult to estimate, improved estimates of the economic burden on society should inform the decision making in two useful ways. First, the estimate gives an idea of the conditions and the populations for which the burden of disease is greatest and can give guidance as to where research on developing new interventions might be focused to provide the greatest potential gain. Furthermore, the detailed estimates of cost components can provide useful input to a cost-effectiveness analysis of a proposed specific intervention, and to its subsequent evaluation. A limited number of international publications have confirmed that the economic repercussions of suicidal behaviors are substantial, with the greatest burden attributed to indirect costs, such as loss of productivity due to work absenteeism and early death [10]. However, the literature on the cost of suicide remains scarce, and although suicide does not always occur following an episode of mental health problems and is a risk across different mental health problems, the more recent publications have considered suicide as an outcome of depression [11, 12]. The most recent estimate of the economic costs of suicide and non-fatal self-harm in the US averaged $510 billion (2020 USD) annually [10]. A study from France [13] which estimated costs of suicide (11,558 cases) and suicide attempts (200,000 cases) in 2019, presented societal costs of EUR 18.5 billion and EUR 5.4 billion for suicides and suicide attempts respectively.

A recently published study presented indirect costs of suicide in Sweden [14], estimated at €44 million over a 1-year horizon and €935 million over a lifetime horizon. Other studies from Sweden have highlighted expected economic consequences generated by people bereaved by suicide. Parents who lost a child to suicide or an accident were shown to have over tenfold higher risk for psychiatric sickness absence exceeding 30 days as compared to non-decedent parents [15]. Likewise, youth self-harm was associated with parents taking approximately a threefold increase in family leave [16]. Conversely, children who lost their parent/caregiver to suicide may be at an increased risk of problems at school, health issues and future work problems [17, 18]. To the best of our knowledge, there are no scientific publications examining the broad economic impact of suicide and self-harm in Sweden. Previous Swedish research has focused either on selected cost components (such as indirect costs alone, or on specific subgroups) leaving the full societal burden unquantified. International estimates, while informative, are based on healthcare systems, labour markets, and coding practices that differ substantially from those in Sweden and therefore cannot be directly applied. This underscores a clear need for a comprehensive, context-specific assessment. Sweden’s extensive national population-based registries provide a unique opportunity to generate robust estimates across multiple cost domains, including healthcare utilisation, emergency services, productivity losses, and the monetary value of disability and premature mortality. Leveraging these high-quality data sources, the aim of this study is to estimate the direct and indirect costs of suicidal behaviours in Sweden for the year 2022, complemented by an assessment of the burden of disease using publicly available data.

## Methods

### Population and epidemiology

This is an incidence-based study, estimating the cost and burden of suicide and self-harm from a societal perspective. The populations considered for the cost calculations were completed suicides and self-harm over a one-year period in 2022. For this study, we used two groups of ICD-10 codes related to suicide and self-harm that are reported in publicly available databases: “Intentional harm” (code: X60-X84, certain suicide), in which the intention of committing suicide is certain and, “Event of undetermined intent” (code: Y10-Y34, uncertain suicide), where a self-injury cannot be clearly distinguished by an accident. We included events of undetermined intent (presented separately) based on the assumption that a substantial proportion of these are suicides. Self-harm was operationalized using the same ICD codes (X60-X84 and Y10-Y34) and were considered certain self-harm for X-diagnoses and uncertain for Y-diagnoses. Data on mortality and number of deaths by suicide were collected from the National Cause of Death register [4] (Swedish National Board of Health and Welfare) while data on healthcare visits (inpatient and outpatient) registered as X60-X84 and Y10-Y34 as primary diagnose were collected from the National Board of Health and Welfare´s data base on injuries and poisonings [19].

In 2022, 1,254 deaths of certain suicide and 315 deaths of uncertain suicide were registered. Additionally, 7,397 patients were admitted to inpatient care (6,392 for certain self-harm and 995 for uncertain self-harm), consuming 28,392 and 4,503 hospital days respectively. Further, 11,523 patients visited physicians in specialized healthcare (4,857 for certain self-harm and 6,66 for uncertain self-harm). From the available data it was not possible to identify which case of inpatient care ends in death. Based on national [20] and international estimations [13, 21], we assumed that only 4% of inpatient care patients admitted with diagnosis X60-X84 and Y10-Y34 died and 96% were self-harm (while all patients in outpatient care were considered as self-harm, thus resulting in total of 18,615 patients of self-harm (10,993 for certain and 7,621 for uncertain self-harm) in 2022. From the available data it was not possible to identify multiple admissions to inpatient and outpatient care. We thus assumed that every patient diagnosed with self-harm had only one admission within each respective care level.

### Costing approach

We combined top-down and bottom-up approaches. Depending on the available data, we used a combination of publicly available information, supplemented by expert opinion. All unit costs were calculated in in Swedish krona and then converted to Euro 2022. We estimated healthcare and non-healthcare direct costs during 2022 as well as indirect costs related to premature death from suicide, consisting of productivity losses from paid and unpaid housework.

### Direct costs

Direct costs comprised of healthcare and non-healthcare costs. Healthcare costs included *forensic examination*, *ambulance transport*,* inpatient care* and *specialized outpatient care*.

The cost of *forensic examination* was sourced from the Forensic Medicine Agency which stated that the cost of a forensic autopsy was 34,400 kronor (€3,234) in 2022 [22]. According to the Swedish Civil Contingencies Agency, about 95% of suicides go through a forensic autopsy [20]. The cost of *ambulance transport* was estimated at 4,892 kronor (€207), based on a national report [23]. In this case, there is controversial evidence on what proportion of the suicide/self-harm actually uses ambulance services. The percentage of patients presenting with self-harm who are transported by ambulance to hospitals varies across studies. One study from England reported an 87% conveyance rate for self-harm cases attended by ambulance services [24]. In contrast, a national study in Scotland found that 23% of patients were admitted to hospitals after ambulance attendance for self-harm [25]. Based on these results, we assumed that 30% would have been transported by ambulance in case of suicide and 80% in case of self-harm. Costs for *inpatient care* were calculated by multiplying the number of days in the hospital due to suicide/self-harm by the daily cost of hospitalization. Amount of days was sourced from the National Board of Health and Welfare´s database of external causes of injury and poisoning [19]. As was mentioned earlier, 4% of the costs for inpatient care were assigned to complete suicides and 96% for self-harm cases. The cost of hospitalization was estimated at 20,558 kronor (€1,933) per day, according to the Cost Per Patient (CPP) database [26]. The cost of a *specialized outpatient care* visit was estimated at 4,463 kronor (€420), according to the CPP database [26]. The number of visits to outpatient care was sourced from the National Board of Health and Welfare´s database [19]. To get an estimate of the cost of specialized outpatient care we multiplied the number of visits by the average cost of a visit. All costs for outpatient care were assigned to self-harm because those patients were alive.

Non-healthcare costs included *police and rescue-*services and *property damage* related to a suicide case. We estimated the costs of attending a suicide by the police and rescue services for 20% and 75% of the registered fatal suicide case respectively. The cost of an intervention from the police was estimated at 1,522 kronor (€143) and for emergency services at 3,037 kronor (€,286) according to a Swedish Civil Contingencies Agency [23] and adjusted to 2022 prices. A proportion of suicides take place in road traffic, where there might be *damage to public or private property* related to the event. According to Swedish Transport Administration (Trafikverket), the cost of property damage related to an accident where a human life was lost is estimated at 17,466 kronor (€1,645) per event [27], adjusted to 2022. It is assumed that 20% of suicides convey property damage in the public roads, according to Swedish Civil Contingencies Agency [20].

### Indirect costs

The indirect costs were measured in terms of productivity loss using the human capital approach [28]. This method considers the individual´s future contribution to production in society if they had continued to work the average capacity of the workforce until retirement. To estimate the productivity losses from *paid work* attributable to suicide, we used the cumulative number of life years lost between the average age of suicide for those who died between the age of 20 years and 65 years because the average retirement age in Sweden in 2022 was 64.8 years [29]. For those who died before 20 years of age we calculate the cumulative number of life years lost between 20 and 65 years. Average annual income (including social fees of 50.5% for 2022) per age group and sex was sourced from Statistics Sweden [30] and then multiplied by the number of lost working years for each age group. The results were then multiplied by the proportion of the population in the workforce that work full time (69% for in 2022 [31]). To estimate the present value of future income lost, a discount rate of 3% was employed, as according Swedish praxis [32]. Additionally, productivity loss calculations considered future annual GDP growth [33]. For this study, we employed a growth factor of 2%. A detailed description of the calculation is presented as an Appendix under the heading ‘Calculation of productivity losses from employment’.

To estimate the productivity losses from *unpaid housework* we used the cumulative number of years lost between the ages of 20 years and 85 years. We valued housework according to the market value principle [33] using average earnings for similar occupations, such as cleaners, care workers and restaurant and kitchen assistants in the labour market.

The unpaid work (work at home) was valued at 244 kronor (€23) per hour regardless of sex and age. This estimation was based on average incomes for occupations similar to household work, such as cleaners, other care and welfare staff, restaurant and kitchen assistants. Time estimates for unpaid work at home for different ages and genders was based on results from a Time Use Survey 2010/11 from Statistics Sweden [34]. An annual discount rate of 3% and growth factor of 2% were employed. Detailed calculations are presented as an Appendix under the heading ‘Calculation of productivity losses from housework’.

### Monetary value of death and disability

The monetary value of death and disability (intangible costs) represents an economic quantification of premature mortality and non-fatal health loss [35]. In this study, the monetary value of death and disability was estimated using DALYs [36]. DALYs were monetized using a human capital approach, valuing one DALY at 1 × GDP per capita. This approach follows historical WHO-CHOICE guidance, which classified interventions costing less than one GDP per capita per DALY averted as “very cost-effective.” Although GDP-based thresholds are no longer formally recommended for priority setting, they remain widely used in burden-of-disease and cost-of-illness studies to facilitate international comparability.

The cost of Disability Adjusted Life Years (DALY)s was calculated based on the Global Burden of Disease and Injury (GBD) report. We valued one DALY based on the 2022 GPD per capita (€45,030 ) [37]. The Global Health Data Exchange (GHDx) data allowed us to estimate how many YLLs that was accounted by suicides as well as the amount of YLDs accounted for self-harm [38].

A summary of the unit costs, assumptions and sources are presented in Table 1.

**Table 1:** Unit costs and percentage of cases used respective services

Societal services costs	Suicide *N* = 1,569 cases (1,254 certain and 315 uncertain), % of cases	Self-harm *N* = 18,615 patients (10,993 certain and 7,621 uncertain), % of patients	Unit	Cost (Euro,2022)	Source
Healthcare costs:					
Direct costs					
*Forensic examination*	95%	NA	Case	3,440	[22]
*Ambulance*	30%	80%	Patient	489	[23]
*Inpatient care*	4%	96%	Admission (day)	2,056	[26]
*Specialized outpatient care*	NA	100%	Visit	446	[26]
Non-healthcare costs:					
*Police*	20%	NA	Case	152	[23]
*Rescue services*	75%	NA	Case	304	[23]
*Property damage*	20%	NA	Case	1,747	[27]
Indirect costs					
*Productivity loss from paid work*	100%	NA	Average annual income by sex and age group		[30]
*Productivity loss from unpaid work (work at home)*	100%	NA	One hour of unpaid work	25	[30], [34]
Death and disability	100%	100%	DALY	45,030	[37]

*NA* not applicable

### Statistical approach and sensitivity analysis

All cost calculations were based on deterministic point estimates and were adjusted for age and sex when estimating productivity losses from both paid and unpaid work.

In order to assess possible uncertainties related to assumptions made in the analyses, one-way sensitivity analyses for costs of suicide were conducted. We calculated the impact on total cost omitting the uncertain suicides (code: Y10-Y34) and only including certain suicides and self-harm (ICD10: X60-X84). Additionally, we varied the discount rate from 3% to 5%, wage growth factor (from 2% to 3%) as well as assumptions for different service usage, such as ambulance (changed from 30% to 80% for cases of suicide) and inpatient care (changed from 4% to 10% for cases of suicide).

## Results

### Direct healthcare costs

In Sweden, a total of 1,569 persons died in suicide (1,254 defined as certain suicide case and 315 as uncertain suicide case) in 2022 and 70% those who died by of suicide were males. As estimated, the majority of suicide death (95%, 1,490 deaths) were subjects for forensic examination. In the same year, 10,481 admissions to inpatient care (9,376 with diagnose X60-X84 and 1,105 with diagnose Y10-Y34) were observed for 7,383 patients. This resulted in a total of 32,843 inpatient care days (28,325 days with diagnosis X60-X84 and 4,518 days with diagnosis Y10-Y34). We proportionally divided costs for inpatient care between suicides (4%) and self-harm (96%). Additionally, 11,471 patients visited specialised outpatient care, and those costs were fully attributed to self-harm. The majority (60%) of inpatient and outpatient patients were females. Direct healthcare costs amounted to €80,771,639 in total, €8,058,673 for suicide and €72,712,966 for self-harm (corresponding to 10% and 90% of total direct healthcare costs, respectively), with a majority of costs falling on inpatient care, 85%, see Table 2.

**Table 2:** Direct, indirect costs and death and disability costs related to suicide and self-harm in Sweden in 2022 (in thousand Euros 2022)

	Suicide (*N* = 1,569)			Self-harm (*N* = 18,615)			Total
	X60-X84 (*N* = 1,254)	Y10-Y34 (*N* = 315)	Total suicide	X60-X84 (*N* = 10,993)	Y10-Y34 (*N* = 7,622)	Total self-harm	
*Direct costs*							
Direct healthcare costs							
Forensic examination	4,098	1,029	5,127				5,127
Ambulance	184	46	230	2,399	374	2,773	3,003
Inpatient care	2,329	372	2,701	55,907	8,917	64,824	67,525
Specialized outpatient care				2,185	2,932	5,116	5,116
Total direct healthcare costs	**6**,**611**	**1**,**447**	**8**,**059**	**60**,**490**	**12**,**223**	**72**,**713**	**80**,**772**
*Direct non-healthcare costs*							
Police	143	36	179				179
Rescue services	76	19	95				95
Property damage	438	110	548				548
Total direct costs	**7**,**269**	**1**,**612**	**8**,**881**	**60**,**490**	**12**,**223**	**72**,**713**	**81**,**594**
*Indirect costs*							
Productivity loss from paid work	517,260	134,971	652,232				652,232
Productivity loss from unpaid work (work at home)	187,568	48,728	236,296				236,296
Total indirect costs	**704**,**828**	**183**,**700**	**888**,**528**				**888**,**528**
Total costs	**712**,** 097**	185,312	**897**,**409**	**60**,**490**	**12**,**223**	**72**,**713**	**970**,**122**
Death and disability monetary value			**2**,**641**,**204**			**61**,**866**	**2**,**703**,**070**

### Direct non-healthcare costs

All direct non-healthcare costs were fully attributed to suicide according to assumptions described in the Methods-section. Direct non-healthcare costs were estimated to be €822,470, with costs related to property damage was the main contributor (70%; property damage 67%, police involvement 22%, rescue services 12% of total non-healthcare costs).

### Indirect costs

#### Loss of productivity from paid work

The loss of productivity from paid work due to suicide was estimated to 25,020 years in 2022, including 20% attributed by uncertain suicides. The total loss of productivity amounted to €652,231,648 (approximately 73% of all indirect costs), including €519,475,921 for males and €164,672,673 for females, see Table 2. The detailed presentation of the results for loss of productivity is presented in Appendix ‘Calculation of productivity losses from employment’.

#### Loss of productivity from unpaid housework (housework, caring, volunteering)

The loss of productivity from unpaid work due to suicide was estimated at 45,411 years in 2022. The total loss of productivity from unpaid housework amounted to € 236,295,983 including € 144,019,660 for males and € 75,143,689 for females, see Table 2. The detailed presentation of the calculation for loss of productivity is presented in Appendix, ‘Calculation of productivity losses from unpaid work at home’.

### Monetary value of death and disability

The GHDx data allowed us to estimate that suicides accounted for 49,796 YLLs while self-harm resulted in 1,166 YLDs and 50, 963 DALYs. In monetary terms, this resulted in a total of €2,7 billion due to disability and lost lives, with suicides accounting for approximately 98% and self-harm for 2% of total DALY-related costs (see Table 2).

#### Total cost of illness and burden of suicide and self-harm in Sweden

The total economic impact of suicides and self-harm occurring during a one-year period was estimated at €970.122 million, of which direct costs represented 8% and indirect costs 92%. Monetary value of death and disability was estimated as €2,7 billion. Tables 2 and 3 present a breakdown of total and average costs, respectively. Based on Sweden’s total population of 10.4 million, the direct costs were calculated at €0.86 per capita for suicides and €6.84 per capita for self-harm. The indirect costs amounted to €85.6 per capita for suicides, while the monetary value of death and disability were €254 per capita for suicides and €6 per capita for self-harm. The average cost of suicide and self-harm (per case) are presented in Table 3 and amounted to €571,862 per case of suicide and €3,813 per case of self-harm.

**Table 3:** The average cost (per case) of suicide and self-harm in Sweden in 2022 (in Euro 2022)

	Suicide (*N* = 1,569)	Self-harm (*N* = 18,615)
Direct costs	5,560	3,813
Indirect costs	566,302	
Productivity loss from paid work	415,699	
Productivity loss from unpaid work (work at home)	150,603	
Total costs	571, 862	3,813
Death and disability monetary value	1,683,368	3,323

### Sensitivity analyses

Results of the sensitivity analyses are presented in Table 4. As expected, increasing the discount rate reduced the total indirect costs from €888.5 million to €524.9 million. Conversely, increasing the growth factor from 2% to 3% raised the total indirect costs from €888.5 million to €1,190.8 million. A higher assumed proportion of ambulance use had no significant impact on total direct healthcare costs, whereas increasing the proportion of inpatient care associated with fatal suicide cases raised total direct healthcare costs from €8.9 million to €12.9 million. However, this change did not substantially alter the share of direct costs in relation to the total societal costs of suicide.

**Table 4:** Results of sensitivity analyses. Direct, indirect costs and death and disability costs related to suicide in Sweden in 2022 (in thousand Euros 2022)

	Base case	Parameters			
		Discounting changed from 3% to 5%	Grow factor changed from 2% to 3%	Use of ambulance changed from 30% to 80%	Use of inpatient care changed from 4% to 10%
*Direct costs*					
Direct healthcare costs					
Forensic examination	5,127	5,127	5,127	5,127	5,127
Ambulance	230	230	230	613	230
Inpatient care	2,701	2,701	2,701	2,701	6,753
Specialized outpatient care					
Total direct healthcare costs	8,059	8,059	8,059	8,442	12,110
Direct non-healthcare costs					
Police	179	179	179	179	179
Rescue services	95	95	95	95	95
Property damage	548	548	548	548	548
Total direct costs	**8**,**881**	**8**,**881**	**8**,**881**	**9**,**264**	**12**,**933**
*Indirect costs*					
Productivity loss from paid work	652,232	405,431	844,471	652,232	652,232
Productivity loss from unpaid work (work at home)	236,296	119,434	346,306	236,296	236,296
Total indirect costs	**888**,**528**	**524**,**866**	**1**,**190**,**777**	**888**,**528**	**888**,**528**
Total costs	**897**,**409**	**533**,**747**	**1**,**199**,**658**	**897**,**792**	**901**,**461**

When only certain cases of suicide and self-harm were included, the total direct and indirect costs of suicide were approximately 20% lower, while the total direct costs related to self-harm were about 40% lower (see Table 2).

## Discussion

To our knowledge, this is the first study to assess the societal cost of suicide, self-harm and the monetary value of suicide-related death and disability in Sweden. Our assessment of the societal costs associated with suicide and self-harm amounted to approximately €970 million in 2022, including direct costs (8%) and indirect costs (92%). The average cost per capita was €93.28. The monetary value related to disability and premature death was estimated at €2.7 billion. Our findings add valuable insights to the existing literature. Only one previous study estimating the indirect costs of suicide in Swedish society based their calculation on data between 2010 and 2019 and this study did not estimate neither direct cost of suicide nor costs of self-harm [14]. According to the findings, the productivity loss from paid work due to suicide was estimated at €935 million over a lifetime horizon which is 30% higher than our estimation, amounting to €625 million. This difference can be explained by following reasons: First, the retirement age in our study was lower, 65 years compared with 67 years, second, we have not included taxes on salary compensation and third, we used incidence data from another time period. On the other hand, the magnitude of the estimations is similar.

International comparisons are difficult due to variations in methodology and data sources, which include different definitions and estimations of cost components. Despite methodological differences, our findings were consistent with other countries with similar healthcare systems when adjusted for population differences. The recently published study by Segar et al. [13] estimated costs of suicide and suicide attempts in France where they reported a weighted average of €355 per capita including a monetary value for death and disability, of which 9.6€ were direct costs, which is in line with our calculations as well as the proportion between direct and indirect costs (7% vs. 93%). In the US, societal costs of suicide and suicide attempts were estimated at $298 per capita, which excluded the value of suicide-related death and disability (3% of direct costs and 97% of indirect costs) [21]. A study from Finland estimated costs related to death of suicide over a lifetime perspective between €456,369 and €309,110 for a 20-year-old and 50-year-old person respectively [39]. These numbers cannot be compared with our results because of different methods used in the estimations. Specifically, Solin and colleagues [39] included loss of labor input as a tax return, costs directly following a suicide, such as forensic examination and costs associated with family members. Further, productivity losses attributed to suicide death were estimated in 28 European Union states for 2015 [40]. According to the results, the productivity losses from paid work in Sweden were €285,394,000 in 2015, which is approximately 44% lower than our estimations for clear suicides in 2022. This difference is largely explained by the lower number of deaths included in the 2015 estimations, 873 compared with 1,254 certain cases in our study. However, productivity losses per suicide case reported in [40] were very close to our estimation, €327,070 versus €412,488. These previous studies have emphasized the need for studies based on reliable national data.

This study has several notable strengths. First, it utilizes data from high-quality national registries, namely the “National cause of death” and “National Patient” registers, thus ensuring that all suicide and self-harm cases diagnosed in the publicly reimbursed healthcare system in Sweden were captured. This is where a large majority of healthcare is provided in Sweden [41]. Second, we used national data to define all unit costs included in the analysis. Third, the study adopts conservative assumptions, suggesting that the estimated costs are likely an underestimation of the true economic burden. Additionally, we used a comprehensive approach to estimate the economic burden of suicide and self-harm, including medical care costs, other direct costs, productivity losses from paid and unpaid housework (indirect costs) and cost of non-monetary burden associated with suicide and self-harm (intangible costs).

Although our study provides valuable insights into the economic burden of suicide in Sweden, it is important to acknowledge certain limitations that may influence the interpretation of our findings. One of the limitations was the use of the human capital method to estimate productivity losses, which may inadvertently lead to an overestimation of the economic impact. Further, summing up productivity losses from paid and unpaid homework may lead to some overestimation due to potential overlap in time use. Another significant limitation concerns the calculation of healthcare costs related to suicide cases, as the available data do not allow for a clear distinction between care provided for suicide diagnoses that result in death (suicide) and those that do not (self-harm/suicide attempts). Additionally, we could not get number of visits in specialized care for patients diagnosed as self-harm/suicide attempts, thus assuming only one visit per patient. Further, we had no reliable data on the proportion of inpatient care among suicide/self-harm. The outpatient care assumption likely led to an underestimation of the true costs of outpatient care for these patients, while the assumption regarding inpatient care was tested in sensitivity analyses and had a relatively small impact on total costs. We can also note major limitations regarding the estimation of societal costs associated with self-harm. We were only able to determine the direct costs, primarily those related to healthcare services. However, it is well known that patients recorded as having attempted suicide/self-harm are often on sick leave and require extensive psychological treatment, medication, and rehabilitation. The group-level data used in this study did not provide access to such detailed information. Furthermore, although we captured a large majority of self-harm attempts resulting in health care visits, many episodes of self-harm never come to medical attention, leading to an underestimation of the economic impact self-harm. Moreover, we were unable to account for the societal costs borne by family members in connection with suicide, which may be substantial. The use of GDP per capita to monetize DALYs has several limitations, particularly in high-income countries such as Sweden. First, GDP per capita reflects average economic productivity rather than societal willingness to pay for health gains and may therefore underestimate or overestimate the true social value of a healthy life year. Second, GDP-based valuation does not account for opportunity costs within the health care system, which are central to priority setting in tax-funded systems like Sweden’s. Third, it assumes uniform economic value across population groups, potentially undervaluing health losses among children, older adults, or individuals outside the labor market. Finally, the historical WHO-CHOICE GDP thresholds were designed as heuristic benchmarks rather than normative decision rules, and they have since been superseded by context-specific and opportunity-cost–based approaches. Consequently, while the 1× GDP benchmark enhances international comparability, results should be interpreted with caution in high-income settings.

A particular challenge in our study was the uncertainty related to Y diagnoses for suicides and self-harm. While using only X diagnoses would certainly underestimate the incidence of suicide and self-harm, including Y diagnoses likely compensates for this underestimation. However, since Y diagnoses comprise both suicides and accidents in unknown proportions, it remains unclear whether this approach overcompensates. We therefore present results for the diagnoses combined and separated. It is important for various stakeholders and policymakers to be aware of the potential variation arising from these assumptions and to consider how it may affect the total cost estimates.

To overcome the aforementioned limitations, a comprehensive register-based study where individual-level data from several national and quality registries were linked, would be desirable. Such a study should include detailed and context-specific information on various societal services related to both confirmed and unconfirmed suicide cases, including those concerning bereaved individuals. If using a matched reference population for such a study, excess costs related to other care received by individuals attempting or committing suicide, not just care where the primary diagnose code is related to suicide, may be estimated.

The results of this study are important for several societal sectors. For example, employers can use our findings to advocate for prevention and promoting mental health programs in the workplace, offering well-being programs, and fostering a supportive environment. Such initiatives can help reduce productivity losses and other indirect costs related to suicide and self-harm. Patient representatives can also use these findings to advocate for stronger mental health services and prevention programs. Highlighting the economic impact of suicidal behavior can strengthen their case to policymakers and raise public awareness of its broader societal costs, encouraging greater community engagement in prevention efforts. Additionally, our findings are necessary to estimate the potential health and economic impact of implementing preventive interventions for suicidal behaviors in the Swedish setting and thus support decision-makers to provide better decisions regarding which interventions provide the most value for money, which, in turn, contributes to a better allocation of limited societal resources. Beyond the implications for economic evaluation, our findings also point to important considerations for health system planning. The scale of the societal burden highlights the need to strengthen crisis intervention capacity, ensure timely access to mental health care, and provide structured postvention support for those affected by suicide. Incorporating these cost estimates into strategic planning can help health services prioritize preventive measures that reduce both immediate harm and longer-term consequences at the population level.

## Conclusion

This study provides the first assessment of the economic burden of suicides and self-harm in Sweden. These findings should prompt policymakers to prioritize strengthening suicide prevention efforts and to support more research aimed at reducing this preventable burden. Further research is necessary to produce a comprehensive estimation of societal costs connected to suicidal behaviors using connected individual-level data.

## Supplementary Information

Supplementary Material 1.

## Data Availability

The data that support the findings of this study are available from the corresponding author, upon reasonable request.
