# Supplementary material for: The economic burden and costs of suicide and self-harm in Sweden
Source: BMC Public Health. 2026 Apr 7;26:1223. doi: 10.1186/s12889-026-27156-z (PMC13081321; doi:10.1186/s12889-026-27156-z)
Supplement: Supplementary file 1 — Supplementary Material 1. [file 12889_2026_27156_MOESM1_ESM.docx]

# Appendix

## Calculation of loss of productivity from employment

Below is a step-by-step explanation of how the productivity loss has been calculated.

Step 1. Number of deaths: Identification of the number of suicide-related deaths by age and sex in the working population (20–64 years), see Table A1

Table A1. Number of deaths i suicide (certain and uncertain) i Sweden, year 2022

| Age | Men | Women | Both |
| --- | --- | --- | --- |
| 20–24 | 53 | 16 | 69 |
| 25–29 | 62 | 21 | 83 |
| 30–34 | 63 | 36 | 99 |
| 35–39 | 68 | 24 | 92 |
| 40–44 | 73 | 21 | 94 |
| 45–49 | 61 | 25 | 86 |
| 50–54 | 70 | 28 | 98 |
| 55–59 | 86 | 30 | 116 |
| 60–64 | 83 | 32 | 115 |

Step 2. Calculate the number of lost working years: Estimate the number of years lost due to premature mortality in each age group from the age at death to the retirement age for the corresponding year, in this case 65 years for 2022. To do this, subtract the average age in each age group from 65 years and multiply by the number of deaths, see table A2

Table A2. Number of lost working years

| Age | | Men | Women | Both |
| --- | --- | --- | --- | --- |
| 20 | 24 | 2279 | 688 | 2967 |
| 25 | 29 | 2356 | 798 | 3154 |
| 30 | 34 | 2079 | 1188 | 3267 |
| 35 | 39 | 1904 | 672 | 2576 |
| 40 | 44 | 1679 | 483 | 2162 |
| 45 | 49 | 1098 | 450 | 1548 |
| 50 | 54 | 910 | 364 | 1274 |
| 55 | 59 | 688 | 240 | 928 |
| 60 | 64 | 249 | 96 | 345 |

Step 3. Calculate the productivity loss. Multiply the number of lost working years by the average annual income (including social security fee, 50.5% for 2022, see table A3) per age group and sex. The estimate is then multiplied by the labour force participation rate (69% in 2022). To calculate the present value of future money, a discount rate of 3% is applied and a growth factor of 2% is included [(number of lost years * (average annual income * social security fee)) / (discount rate / growth factor)]. See table A4

Table A3. Average annual income in 2022 (Euro) including social fee (50.5%)

| Age | Men | Women | Both |
| --- | --- | --- | --- |
| 20–24 | 28,722 | 23,280 | 26,001 |
| 25–29 | 44,932 | 37,084 | 41,008 |
| 30–34 | 56,317 | 44,100 | 50,209 |
| 35–39 | 63,387 | 49,038 | 56,213 |
| 40–44 | 70,283 | 55,949 | 63,116 |
| 45–49 | 75,751 | 60,336 | 68,043 |
| 50–54 | 76,422 | 60,214 | 68,318 |
| 55–59 | 72,559 | 57,797 | 65,178 |
| 60–64 | 65,535 | 52,180 | 58,857 |

Source: Statistics Sweden (SCB) [Statistics Sweden](https://www.scb.se/en/)

Table A4. Loss of productivity, certain and uncertain suicide (Euro, 2022)

| Age | Men | Women | Both |
| --- | --- | --- | --- |
| 20–24 | 50,417,139 | 17,707,847 | 65,418,337 |
| 25–29 | 66,679,931 | 20,134,367 | 83,121,665 |
| 30–34 | 73,419,026 | 30,565,310 | 100,254,565 |
| 35–39 | 80,142,982 | 21,628,555 | 95,865,124 |
| 40–44 | 82,881,955 | 19,155,051 | 96,039,009 |
| 45–49 | 62,354,758 | 18,860,416 | 77,279,979 |
| 50–54 | 51,327,008 | 19,507,121 | 68,016,727 |
| 55–59 | 38,156,552 | 12,393,662 | 48,251,589 |
| 60–64 | 14,096,569 | 4,720,344 | 17,984,653 |
| Total | 519,475,921 | 164,672,673 | 652,231,648 |

We have also done separate calculations for certain and uncertain suicide, see table A5 and table A6.

Table A5. Loss of productivity, certain suicide (Euro, 2022)

| Age | Men | Women | Both |
| --- | --- | --- | --- |
| 20–24 | 43,694,854 | 14,983,562 | 56,290,197 |
| 25–29 | 50,416,533 | 14,094,057 | 61,599,091 |
| 30–34 | 58,549,350 | 26,198,838 | 82,026,462 |
| 35–39 | 63,368,870 | 17,302,844 | 76,030,960 |
| 40–44 | 65,057,879 | 14,898,373 | 75,230,557 |
| 45–49 | 48,147,345 | 15,717,013 | 60,973,194 |
| 50–54 | 42,269,301 | 13,321,936 | 52,901,899 |
| 55–59 | 31,858,869 | 8,852,616 | 38,601,271 |
| 60–64 | 10,934,722 | 3,356,689 | 13,606,810 |
| Total | 414,297,722 | 128,725,928 | 517,260,442 |

Table A6. Loss of productivity, uncertain suicide (Euro, 2022)

| Age | Men | Women | Both |
| --- | --- | --- | --- |
| 20–24 | 6,722,285 | 2,724,284 | 9,128,140 |
| 25–29 | 16,263,398 | 6,040,310 | 21,522,574 |
| 30–34 | 14,869,676 | 4,366,473 | 18,228,103 |
| 35–39 | 16,774,113 | 4,325,711 | 19,834,164 |
| 40–44 | 17,824,076 | 4,256,678 | 20,808,452 |
| 45–49 | 14,207,413 | 3,143,403 | 16,306,785 |
| 50–54 | 9,057,707 | 6,185,185 | 15,114,828 |
| 55–59 | 6,297,683 | 3,541,046 | 9,650,318 |
| 60–64 | 3,161,847 | 1,363,655 | 4,377,843 |
| Total | 105,178,199 | 35,946,745 | 134,971,206 |
|  |  |  |  |

## Calculation loss of productivity from unpaid work at home

Below is a step-by-step explanation of how the productivity loss from unpaid work at home
Step 1. To value housework according to the market value principle, the average income for similar occupations in the labour market is used, as shown in table A7. The unpaid work performed by employed individuals is then valued at 23 Euro per hour regardless of gender and age. (3,674 Euro / 20 working days / 8 hours per day = 23 Euro per hour), see table A7.

Table A7. Average incomes for occupations similar to household work, year 2022

| Occupation | Average monthly income (Euro, 2022) |
| --- | --- |
| Cleaners | 2,407 |
| Other care and welfare staff | 2,548 |
| Restaurant and kitchen assistants | 2,369 |
| Average for all occupations | 2,441 |
| Including social fee (50,5%) | 3,674 |

Source: Statistics Sweden (SCB) [Statistics Sweden](https://www.scb.se/en/)

Step 2. The next step would be to multiply the hourly wage for unpaid work by the average amount of time spent on housework per age group and gender (see Table A8). The data on time spent on housework was sourced from Swedish population survey.

Tabell A8. Average time for unpaid homework per day.

| Age | Men (hours) | Women (hours) | Both (Hours) |
| --- | --- | --- | --- |
| 20–64 | 3.0 | 3.8 | 3.4 |
| 65–84 | 4.6 | 3.9 | 4.3 |

*Source: SCB Tidsanvändringsundersökning 2010/11(Time-use survey 2010/2011)*

If we assume that an average person would perform work at home 2 days a week every month over the course of a year (96 days per year), we estimate the annual value of work at home (23 Euro * hours/day) * 96 days).

Tabell A9. Annual value of work at home, Euro 2022

| Age | Men | Women | Both |
| --- | --- | --- | --- |
| 20–64 | 6,614 | 8,377 | 7,496 |
| 65–84 | 10,053 | 8,686 | 9,369 |

Step 3. The final step is to multiply the average annual value of housework by all the lost years for people who died prematurely due to suicide. Discounting and adjustment for the growth factor also applied [(number of lost years * average value of housework) / (discounting / growth factor)]. See Table 10A.

Tabell 10A. Loss of productivity from unpaid work at home, certain and uncertain suicide (Euro, 2022)

| Age | Men | Women | Both |
| --- | --- | --- | --- |
| 20–24 | 13,385,747 | 4,822,281 | 19,668,387 |
| 25–29 | 17,292,335 | 8,214,730 | 27,222,978 |
| 30–34 | 15,872,833 | 11,016,283 | 28,123,566 |
| 35–39 | 16,291,748 | 7,467,700 | 25,533,828 |
| 40–44 | 16,396,433 | 6,306,175 | 24,720,114 |
| 45–49 | 12,749,498 | 6,481,287 | 20,701,396 |
| 50–54 | 12,285,399 | 8,043,728 | 21,636,556 |
| 55–59 | 12,936,181 | 7,299,983 | 21,927,167 |
| 60–64 | 11,170,696 | 6,691,589 | 19,493,477 |
| 65–84 | 15,638,790 | 8,799,932 | 27,268,514 |
| Total | 144,019,660 | 75,143,689 | 236,295,983 |

We have also done separate calculations for certain and uncertain suicide, see table A5 and table A6.

Table A11. Loss of productivity from unpaid work at home, certain (Euro, 2022)

| Age | Men | Women | Both |
| --- | --- | --- | --- |
| 20–24 | 11,630,239 | 4,538,618 | 17,398,958 |
| 25–29 | 13,074,692 | 5,750,311 | 20,174,171 |
| 30–34 | 12,658,082 | 9,442,529 | 23,010,190 |
| 35–39 | 12,881,847 | 5,974,160 | 20,250,967 |
| 40–44 | 12,870,318 | 4,904,803 | 19,364,090 |
| 45–49 | 9,844,549 | 5,401,072 | 16,333,212 |
| 50–54 | 10,117,387 | 5,493,277 | 16,828,433 |
| 55–59 | 10,801,083 | 5,214,274 | 17,541,733 |
| 60–64 | 8,665,120 | 4,758,463 | 14,748,354 |
| 65–84 | 13,062,990 | 6,468,326 | 21,917,435 |
| Total | 115,606,307 | 57,945,833 | 187,567,542 |

Table A12. Loss of productivity from unpaid work at home, uncertain suicide (Euro, 2022)

| Age | Men | Women | Both |
| --- | --- | --- | --- |
| 20–24 | 1,755,508 | 283,664 | 2,269,429 |
| 25–29 | 4,217,643 | 2,464,419 | 7,048,807 |
| 30–34 | 3,214,751 | 1,573,755 | 5,113,376 |
| 35–39 | 3,409,901 | 1,493,540 | 5,282,861 |
| 40–44 | 3,526,115 | 1,401,372 | 5,356,025 |
| 45–49 | 2,904,949 | 1,080,214 | 4,368,184 |
| 50–54 | 2,168,012 | 2,550,450 | 4,808,124 |
| 55–59 | 2,135,098 | 2,085,709 | 4,385,433 |
| 60–64 | 2,505,577 | 1,933,126 | 4,745,123 |
| 65–84 | 2,575,801 | 2,331,606 | 5,351,079 |
| Total | 28,413,353 | 17,197,855 | 48,728,441 |
